# Physicochemical Properties and Hypolipidemic Activity of Soluble Dietary Fiber Extracted from Ginger Peel Residue

**DOI:** 10.3390/foods15152685

**Published:** 2026-07-30

**Authors:** Ming Zhang, Fuxia Hu, Yuyu Zhang, Chao Ma, Mengxue Sun, Maoyu Wu, Li Liang

**Affiliations:** 1 School of Food and Health, Beijing Technology and Business University, Beijing 100048, China; zhangming_101@126.com (M.Z.); zhangyuyu@btbu.edu.cn (Y.Z.); 2 Jinan Fruit Research Institute, All China Federation of Supply and Marketing Co-Operatives, Jinan 250014, China; 18864801380@163.com (F.H.); mch.01@163.com (C.M.); smx18313941107@163.com (M.S.)

**Keywords:** steam explosion, structural characterization, HepG2 cells, zebrafish model, lipid metabolism

## Abstract

Hyperlipidemia is a chronic metabolic disorder driven by impaired lipid homeostasis. In this study, the steam explosion (SE)-assisted extraction was applied to obtain soluble dietary fiber (SDF) from ginger peel residue. Based on single-factor and orthogonal optimization experiments, SE treatment at a material-to-cavity ratio of 2:5 and a pressure of 1.5 MPa for 300 s achieved an SDF extraction yield of 8.20%, representing a 210.60% increase. Structural and physicochemical analyses showed that both SDF and SE-SDF were mainly composed of glucose, galactose and mannose. SE treatment generated a looser and more porous structure and significantly improved the thermal stability, water holding capacity (WHC) and swelling capacity (SC) of SE-SDF increasing by 38.89% and 30.77%, respectively (*p* < 0.05). In oleic acid/palmitic acid (OA/PA)-stimulated HepG2 cells, both SDF and SE-SDF reduced triglyceride (TG) and total cholesterol (TC) levels in a dose-dependent manner, with SE-SDF showing a stronger hypolipidemic activity. Zebrafish experiments further confirmed that 200 µg/mL SE-SDF significantly reduced TG and TC levels by 30.58% and 24.89%, respectively, and decreased lipid deposition in the caudal vasculature and liver by 65.59% and 33.86%, respectively. These findings indicate that SE-SDF from ginger peel residue is a promising functional food ingredient for hyperlipidemia prevention.

## 1. Introduction

Hyperlipidemia is a metabolic syndrome characterized by elevation of blood lipid levels resulting from lipid metabolism disorder. It is typically characterized by elevated serum levels of triglycerides (TG) and total cholesterol (TC) [1,2]. This condition is intrinsically linked to the pathogenesis of atherosclerosis, cardiovascular diseases, non-alcoholic fatty liver, and other chronic metabolic disorders [3,4]. Driven by shifting dietary patterns and lifestyle, the global prevalence of hyperlipidemia has escalated significantly. By 2025, the global number of individuals affected by hyperlipidemia has reached 1.28 billion, accounting for approximately 25.3% of the adult population, marking a substantial surge since 2018 and posing a formidable challenge to global public health [5,6]. At present, statins and fibrates remain the primary clinical treatments, but long-term use is frequently associated with adverse reactions (e.g., gastrointestinal discomfort, liver and kidney function damage) [7]. Therefore, the development of safe and effective dietary intervention strategies to regulate lipid metabolism has attracted considerable attention in food science and nutritional health.

Dietary fiber (DF), often termed the “seventh major nutrient,” plays a critical role in regulating lipid metabolism. Extensive research indicates that DF exerts hypolipidemic effects through multiple mechanisms, such as adsorbing cholesterol, promoting bile acid excretion, delaying lipid absorption, and regulating gut microbiota [8,9]. However, natural DF often suffers from dense structure, poor solubility, and low bioavailability, which limit its functional properties and foods application [10]. Among different types of DF, soluble dietary fiber (SDF) exhibits prominent physiological activity in improving lipid metabolism and intestinal health due to its high water holding capacity (WHC), viscosity, and fermentability [11]. Therefore, modifying the structure of DF through physical or chemical methods to enhance its physicochemical properties and biological activity is a current research hotspot.

At present, DF modification methods mainly include physical, chemical, and biological approaches. Among them, physical modification (e.g., ultrasound-assisted treatment, microwave-assisted treatment, steam explosion (SE) treatment) has attracted considerable attention because of its high modification efficiency, suitability for large-scale production, and the absence of chemical reagent residues [12]. Compared with other physical modification methods, SE integrates high-temperature, high-pressure, and mechanical shear effects, which effectively disrupt the lignocellulosic matrix, promote the conversion of insoluble dietary fiber (IDF) into SDF, and improve the physicochemical properties of SDF [13,14]. For instance, Ma et al. demonstrated that the SE process significantly increased the SDF content in buckwheat bran, while enhancing its thermal stability, antioxidant and antibacterial properties [15]. Similarly, He et al. found that SE increased the SDF content of Tartary buckwheat bran by 54.11% and significantly improved its hydration properties and thermal stability [16].

Ginger, as an important medicinal and edible plant, generates a large amount of peel residue during processing. At present, most ginger peel residue is discarded or utilized as low-value animal feed or fertilizer, leading to resource wastage and environmental concerns. Recent studies have highlighted that ginger peel residue is rich in DF, which exhibits various pharmacological activities, including hypolipidemic, prebiotic, and antioxidant effects [17,18]. It is a functional raw material with potential for development. However, DF is mainly in the form of IDF, with a low content of SDF, which limits its functional properties and application. While SE technology has been applied to various agricultural and forestry by-products, research regarding the structural regulation and functional enhancement of DF from ginger peel residue remains scarce, and its underlying mechanisms have yet to be elucidated.

Therefore, the aim of this study was to investigate whether SE pretreatment could improve the structural characteristics, physicochemical properties, and hypolipidemic activity of SDF extracted from ginger peel residue. SDF extracted after SE pretreatment (SE-SDF) was systematically characterized in terms of its physicochemical and structural properties. Subsequently, the potential hypolipidemic effects of SE-SDF were evaluated in vitro using HepG2 cells. Furthermore, the hypolipidemic effects of SE-SDF were validated in zebrafish model. These findings provide a scientific basis for utilizing ginger peel derived SDF as a functional food ingredient for the dietary management of hyperlipidemia.

## 2. Materials and Methods

### 2.1. Materials and Reagents

Ginger peel residue was obtained from Anqiu Linfu Food Co., Ltd. (Weifang, China). Anhydrous ethanol, anhydrous sodium carbonate and potassium bromide were purchased from Damao Chemical Reagent Co., Ltd. (Tianjin, China); α-Amylase (40,000 U/g, derived from *Aspergillus oryzae*), neutral protease (40,000 U/g, derived from *Bacillus subtilis*), Folin–Ciocalteu reagent were purchased from Soleibao Technology Co., Ltd. (Beijing, China); gallic acid, 2,2-diphenyl-1-picrylhydrazyl (DPPH) and 2,2′-Azino-bis(3-ethylbenzothiazoline-6-sulfonic acid) (ABTS) were purchased from Hualan Chemical Technology Co., Ltd. (Shanghai, China); Cell Counting Kit-8 (CCK-8), TG, TC, oleic acid/palmitic acid (OA/PA) were all purchased from Jiancheng Biotechnology Co., Ltd. (Nanjing, China). Egg yolk powder was purchased from Yuanye Biotechnology Co., Ltd. (Shanghai, China); Oil Red O staining reagent was purchased from Merck Life Sciences Ltd. (Nantong, China). All other reagents were analytical grade.

### 2.2. SE Pretreatment

A quantity (100 g) of ginger peel residue was exploded in a SE device (QBS-80, Hebi Zhengdao Qibao Industrial Co., Ltd., Hebi, China) under the conditions of material-to-cavity ratio of 3:5, SE pressure of 1.2 MPa, and time of 120 s. Following SE pretreatment, the ginger peel residue was oven-dried at 60 °C to a moisture content below 10%, ground, and passed through a 60-mesh sieve before subsequent experiments.

### 2.3. Extraction of SDF from Ginger Peel Residue

The extraction of SDF from ginger peel residue was based on the enzymatic method of Ma et al. [19] with slight modification. A quantity (5.0 g) of the ginger peel residue (with or without SE pretreatment) was accurately weighed into a 250 mL conical flask and mixed with 150 mL distilled water. Then, 0.3% (*w*/*w*) α-amylase was added and underwent hydrolysis at 90 °C for 30 min using a constant temperature shaker (SHA-B, Jarier Co., Ltd., Jiaxing, China). After 0.3% (*w*/*w*) α-amylase hydrolysis, the sample was inactivated at 95 °C for 5 min. Next, 0.5% (*w*/*w*) neutral protease was introduced for hydrolysis at 50 °C for 60 min, followed by enzyme inactivation at 95 °C for 5 min. Finally, the sample was centrifuged at 4000 rpm for 10 min, and the collected supernatant was concentrated under reduced pressure and precipitated with 4 volumes of anhydrous ethanol solution for 12 h. The precipitate was recovered by centrifugation, freeze-dried, and used as the SDF sample. The extraction rate was determined using the following equation:

Extraction rate (%) = (m_1_/m_2_) × 100, (1)

where m_1_ is the mass of SDF obtained after freeze-drying (g); m_2_ is the mass of ginger peel residue sample (g).

### 2.4. Single-Factor Experiment

The single-factor experiment was performed to evaluate the effects of SE parameters on the SDF extraction of ginger peel residue under different conditions: material-to-cavity ratio (1:5, 2:5, 3:5, 4:5, 4:5), SE pressure (0.3, 0.6, 0.9, 1.2, 1.5, 1.8 MPa) and SE time (60, 120, 180, 240, 300 s).

### 2.5. Orthogonal Experiment

Following the single-factor experiments, material-to-cavity ratio (A), SE pressure (B), and SE time (C) were further optimized using a three-factor, three-level orthogonal design (Table 1).

### 2.6. Determination of Chemical Components of SDF and SE-SDF from Ginger Peel Residue

The polysaccharide content of SDF and SE-SDF was determined according to the phenol-sulfuric acid method, in which carbohydrates react with phenol and sulfuric acid to form colored compounds that were quantified spectrophotometrically [20]. Polyphenol content was determined by the Folin–Ciocalteu colorimetric method [21]. Protein content was measured using the Bradford assay as previously reported [22].

### 2.7. Structure Analysis of SDF and SE-SDF from Ginger Peel Residue

#### 2.7.1. Determination of Monosaccharide Composition

The monosaccharide composition was determined according to a previous method with minor modification [23]. The monosaccharide standards (Glucose, Galactose, Arabinose, Rhamnose, Fucose, Mannose, Ribose, Xylose, Glucuronic acid (GluUA) and galacturonic acid (GalUA)), SDF and SE-SDF were subjected to pre column derivatization and collected into a sample bottle for high-performance liquid chromatography (HPLC) analysis. HPLC analysis was performed with a Shimadzu LC-20AR analytical liquid chromatography system (Shimadzu, Kyoto, Japan), equipped with a Uranus 5u C18 column (4.6 mm × 250 mm, 5 μm). The mobile phases consist of acetonitrile (A) and 0.02 mol/L ammonium acetate (B); The flow rate was 1.0 mL/min with the column temperature at 30 °C, and detection wavelength at 254 nm with the VWD detector.

#### 2.7.2. Determination of Molecular Weight (Mw)

The Mw of SDF and SE-SDF was verified by high-performance gel permeation chromatography (HPGPC) using a Shimadzu RID-20A differential detector in the Shimadzu LC-20AR analytical liquid chromatography system (Shimadzu, Kyoto, Japan) coupled with TSK gel GMPWXL (300 mm × 7.8 mm, TosoHaas Corp., Shunan, Japan) column in series. The analysis was conducted using ultrapure water as the eluent at a flow rate of 0.6 mL/min, while the column was maintained at 35 °C [24].

#### 2.7.3. Determination of Fourier Infrared Spectroscopy (FT-IR)

The FT-IR of SDF and SE-SDF was determined according to Zhang et al. [25] with some modifications. The samples were mixed with 200 mg KBr, pressed into a 1 mm thick flake, and then used for machine detection. The IR spectra was recorded in the range of 4000–4450 cm^−1^ and 32 scans using a Nicolet iZ-10 Fourier Transform Infrared Spectrometer (Thermo, Waltham, MA, USA) with a DTGS detector.

#### 2.7.4. Determination of Thermal Stability Analysis

The thermal stability of SDF and SE-SDF was determined by thermogravimetric analysis (TGA). TGA analysis was performed under a nitrogen atmosphere, with a temperature range of 25–1100 °C and a heating rate of 10 °C/min [26]. The thermogravimetric curve and weight loss rate curve of the sample were recorded to evaluate its thermal stability.

#### 2.7.5. Determination of Scanning Electron Microscopy (SEM)

The samples were propagated onto conductive adhesive, treated with gold spray, and observed using a JCM-7000 NeoScope scanning electron microscope (NeoScope, Tokyo, Japan).

### 2.8. Physicochemical Analysis of SDF and SE-SDF from Ginger Peel Residue

#### 2.8.1. Determination of WHC

The determination of WHC was carried out, following a published method with slight modification [27]. A quantity (0.5 g) of SDF and SE-SDF was suspended in 10 mL of deionized water and allowed to stand at room temperature for 12 h. Following centrifugation (5000 r/min, 20 min), the supernatant was decanted and the remaining hydrated residue was weighed. WHC was determined according to the following equation:

WHC (g/g) = (m_2_ − m_1_)/m_1_, (2)

where m_1_ is the mass of the sample (g); m_2_ is the mass of precipitate (g).

#### 2.8.2. Determination of Oil Holding Capacity (OHC)

OHC was determined according to the method described by Yin et al. [28]. Briefly, 3.0 g of SDF and SE-SDF were weighed and mixed with 40 g of peanut blend oil for 30 min in a 50 mL centrifuge tube. Then, the mixture was centrifuged at 5000 r/min for 20 min, and the supernatant oil was collected. The OHC was calculated according to the following equation:

OHC (g/g) = (40 − m_2_)/m_1_, (3)

where m_1_ is the mass of the sample (g); m_2_ is the mass of supernatant oil (g).

#### 2.8.3. Determination of Swelling Capacity (SC)

1.0 g of SDF and SE-SDF was transferred into a graduated tube, and its initial volume was noted. Distilled water (10 mL) was then added, followed by thorough mixing. After standing at room temperature for 24 h to allow complete swelling, the final volume was measured, and SC was calculated according to the following equation:

SC (mL/g) = (V_2_ − V_1_)/m, (4)

where m is the mass of sample (g); V_1_ is the initial volume of sample (g); V_2_ is the final volume of precipitate (mL).

### 2.9. Determination of Hypolipidemic Effect of SDF and SE-SDF from Ginger Peel Residue in HepG2 Cells

#### 2.9.1. Cell Culture and Cell Viability Analysis

HepG2 cells were obtained from Ponosi Life Technology Co., Ltd. (Wuhan, China). HepG2 cells were cultured in DMEM containing 10% FBS and 1% P/S at 37 °C in a cell humidified incubator with 5% CO_2_. The cells were cultured until they reach 80–90% confluence, and then the cells were respectively seeded into 96-well plates at a density of 5 × 10^4^ cells/well. After 24 h of culture, HepG2 cells were treated with fresh medium containing the different concentrations of SDF, SE-SDF (50, 100, 200, 400 μg/mL), and OA/PA (0.125, 0.25, 0.5, 1 mmol/L) [19,29]. After 24 h of culture, the cell viability was measured using the CCK-8 kit at 450 nm using an enzyme labeling instrument (Bio Tek, Windsor, VT, USA).

#### 2.9.2. TG and TC Content Analysis

HepG2 cells were plated in 6-well plates at a density of 1 × 10^6^ cells per well and incubated for 24 h prior to treatment. ND group (the normal cell culture), model group (HepG2 cells were cultured for 24 h and then treated with 0.125 mmol/L OA/PA for 24 h), and a treatment group (HepG2 cells were pretreated with SDF and SE-SDF (50, 100, 200, 400 µg/mL) and then treated with 0.125 mmol/L OA/PA for 24 h). After cell culture, the cells were washed with PBS, lysed with lysis buffer and centrifuged for 10 min to obtain the supernatant. Then, the TC and TG content were determined using the test kits, respectively.

### 2.10. Determination of Hypolipidemic Effect of SDF and SE-SDF from Ginger Peel Residue in High-Fat Zebrafish

#### 2.10.1. Body Length and Weight Analysis

Zebrafish share highly conserved lipid metabolic pathways with humans, and their transparent larvae allow direct visualization of lipid accumulation, making them a widely used model for lipid metabolism studies [30].

AB series zebrafish were provided by Biology Institute of Shandong Academy of Sciences (Jinan, China). All Zebrafish were raised at 28 ± 2 °C, with 14 h of light exposure and 10 h of darkness, and fed twice/day with prawns. A total of wild-type AB strain zebrafish larvae (15 days post fertilization, 15 dpf) were randomly divided into 8 experimental groups, with ten larvae in each group. ND group: fed with the normal diet; Model group (high-fat diet, HFD): fed with 0.2% egg yolk powder, twice/d; Treatment group: fed with SDF and SE-SDF (50, 100, 200 µg/mL) along with egg yolk powder (0.2%). After 48 h of feeding, all zebrafish were collected, anesthetized, body weighed, and the body length was measured under a Zeiss stereomicroscope (ZEISS, Oberkochen, Germany).

#### 2.10.2. TG and TC Content Analysis

The zebrafish were handled as referred to in Section 2.10.1. After feeding, the zebrafish were washed three times with PBS and added to PBS in a weight (g):volume (mL) ratio of 1:9. Then, the mixture was homogenized under ice bath conditions, and the TG and TC contents were measured according to the reagent kits.

#### 2.10.3. Histopathological Analysis

The Oil red O staining was performed with reference to Zheng et al. [31] and with slight modification. The zebrafish were handled as referred to in Section 2.10.1. Zebrafish were fixed with 4% paraformaldehyde for 4 h for overall Oil red O staining. After staining, zebrafish were fixed with 4.2% methylcellulose, the lipid accumulation in the caudal vasculature and liver regions were observed under a IX73 Olympus inverted microscope (Olympus, Tokyo, Japan) and photographed. Then, the IOD of the caudal vasculature and liver regions of the zebrafish were measured and calculated using Image Pro plus software 7 (Media Cybernetics, Bethesda, MD, USA).

### 2.11. Statistical Analysis

All experiments were performed in triplicate, and the results were presented as the mean ± standard deviation. The date were analyzed by one-way analysis of variance (ANOVA) followed by Tukey’s multiple comparison test using SPSS 26.0 software (IBM Corp., Armonk, NY, USA). The graphs were created using the Prism 10 (GraphPad, La Jolla, CA, USA) and Image Pro-plus 7 (Media Cybernetics, USA).

## 3. Results and Discussion

### 3.1. Effects of the SE Treatment on the Extraction Rate of the SDF

**Figure 1:**
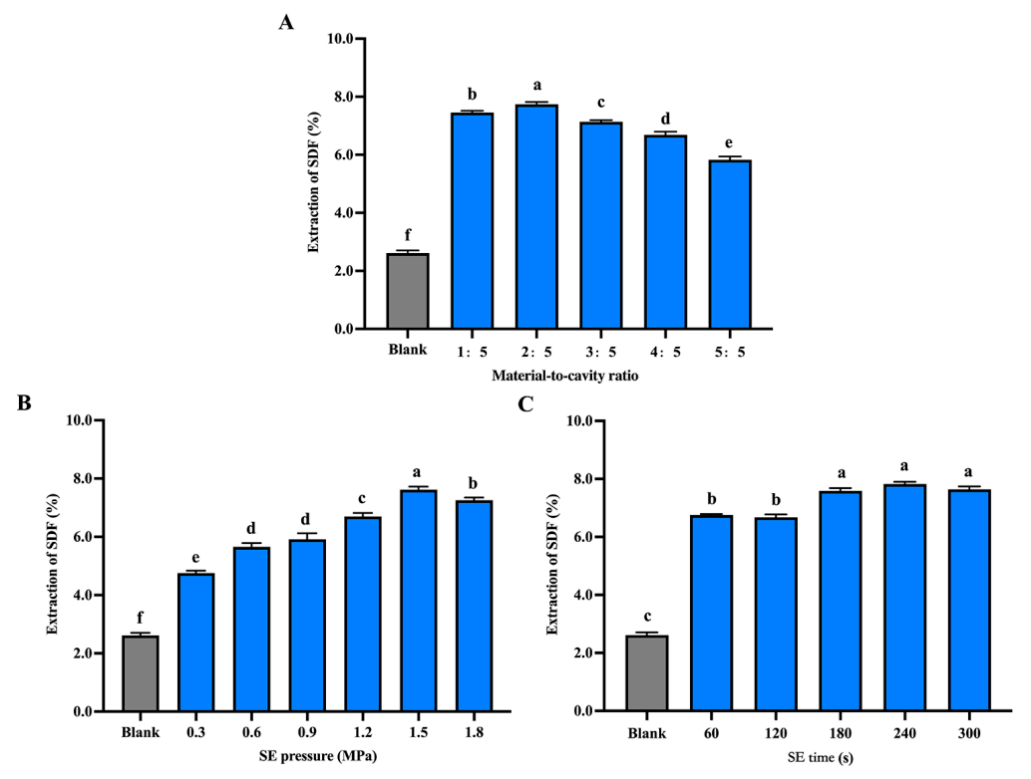
Effects of the different variables on the extraction rate of the SDF and SE-SDF of ginger peel residue: (**A**) material-to-cavity ratio; (**B**) SE pressure; (**C**) SE time. **Note:** Different letters indicate significant differences among groups (*p* < 0.05).

The effects of different material-to-cavity ratio, SE pressure and time on the extraction rate of SDF from ginger peel residue are shown in Figure 1. Among the tested parameters, the material-to-cavity ratio exerted a significant influence on the extraction yield (*p* < 0.05). When the material-to-cavity ratio was 2:5, the extraction rate of SE-SDF reached a maximum of 7.71%, which was approximately 3-fold higher than that of SDF. A high material-to-cavity ratio can promote sufficient contact between high-temperature steam and the feed material, thereby enhancing the steam explosion effect. However, when the material-to-cavity ratio exceeded 2:5, the extraction yield changed slightly. This may be attributed to the limited steam penetration into materials, which weakened the thermomechanical disruption and cell wall disintegration effects, resulting in a decrease in extraction rate. Therefore, a material-to-cavity ratio of 2:5 was selected for the subsequent experiments.

As shown in Figure 1B, the extraction rate of SE-SDF was the highest at an SE pressure of 1.5 MPa. The increase in pressure may lead to the destruction of plant cell walls and accelerate the dissolution of substances such as SDF. However, a further increase in pressure led to a decline in extraction rate. This decrease may be attributed to the fact that excessive pressure induced the degradation of polysaccharides. Part of the soluble polysaccharides was converted into low-molecular-weight polysaccharides or oligosaccharides, making ethanol precipitation more difficult and thereby reducing the SDF extraction rate [32]. A similar result was also found in the study on the SDF from sweet potato residue [33]. Accordingly, the optimal SE pressure was selected as 1.5 MPa. As shown in Figure 1C, the extraction rate increased as the treatment time was extended from 180 to 240 s, but declined when the treatment was further prolonged, with the highest value (7.80%) obtained at 240 s. This may be due to prolonged SE treatment promoting the chemical reactions, such as caramelization and Maillard reactions, leading to the degradation of SDF and a decrease in the extracted SDF content [34]. Therefore, the SE time of 240 s was selected.

### 3.2. Orthogonal Experiment Analysis

The orthogonal experiment results are shown in Table 2. According to the range analysis results, three factors had an impact on the extraction rate of SE-SDF from ginger peel residue: B > A > C, that is, SE pressure > material-to-cavity ratio > SE time. By comparing the k values at different levels, the optimal extraction conditions for the SE-SDF from ginger peel residue were determined to be A2B2C3, that was: material-to-cavity ratio, 2:5; SE pressure, 1.5 MPa; SE time, 300 s. Under these conditions, the extraction rate of SE-SDF from ginger peel residue was 8.20%, which was 210.6% higher than that of the SDF.

### 3.3. Chemical Components of SDF and SE-SDF Extracted from Ginger Peel Residue

As shown in Table 3, the total polysaccharide content of SDF and SE-SDF both remained at a high level (>96%), with no significant difference between them, indicating that SE treatment did not have a significant effect on the overall polysaccharides content. However, SE exerted a significant effect on polysaccharide composition and structure. After SE pretreatment, the neutral polysaccharides content of SDF increased to 89.29 ± 1.46%, representing a 4.6% increase compared with the untreated sample. In addition, the acidic polysaccharides content decreased from 7.91% to 5.40%. These results might be due to the fact that the high temperature and pressure led to (i) the destruction of plant cell walls, enhancing the release of polysaccharides [35] and/or (ii) the cleavage or degradation of uronic acid-containing side chains, reducing polysaccharide branching and increasing the relative proportion of neutral polysaccharides [36]. In addition, SE treatment increased the protein content from 0.38% to 1.49%. The high-pressure thermoacidic environment and strong shear effects disrupted non-covalent interactions and even partial covalent bonds between proteins and polysaccharides, which promoted protein dissolution [37]. Similar findings have been reported previously; the protein from peanut meal imparted an increase with the SE treatment, consistent with the results of this study [38]. Previous studies have shown that derived proteins and peptides can improve lipid metabolism by regulating lipid synthesis and cholesterol metabolism [39]. Therefore, the increased protein content in SE-SDF may also contribute to its enhanced hypolipidemic activity.

### 3.4. Monosaccharide Composition Analysis

The monosaccharide composition and Mw of SDF and SE-SDF were presented in Table 4. Both SDF and SE-SDF were mainly composed of glucose, galactose, xylose and arabinose, among which glucose was the predominant monosaccharide, accounting for 90.36% and 89.05%, respectively. After SE treatment, the glucose content of SDF slightly decreased, while the contents of galactose, xylose, and arabinose all increased by 12.22%, 164.22%, and 59.79%, respectively. The increase in xylose and arabinose may result from the severe physicochemical conditions generated during SE treatment. High temperature and pressure, and rapid depressurization disrupted the cellulose and hemicellulose matrix, facilitating hemicellulose depolymerization and the release of xylose- and arabinose-rich fragments [35]. Previous studies have demonstrated that the biological activity of polysaccharides is closely associated with their monosaccharide composition. For example, Liu et al. reported that a galactose- and arabinose-rich polysaccharide from Suaeda salsa markedly alleviated lipid accumulation in hyperlipidemic zebrafish [40]. In addition, GalUA was not detected in SE-SDF, further indicating that some of the glucuronic acid structures may have degraded during the SE process, which was consistent with the previous chemical composition result. The Mw of SDF decreased to 122.99 kDa after SE treatment, indicating cleavage of polysaccharide chains and a reduction in molecular weight.

### 3.5. FT-IR Analysis

**Figure 2:**
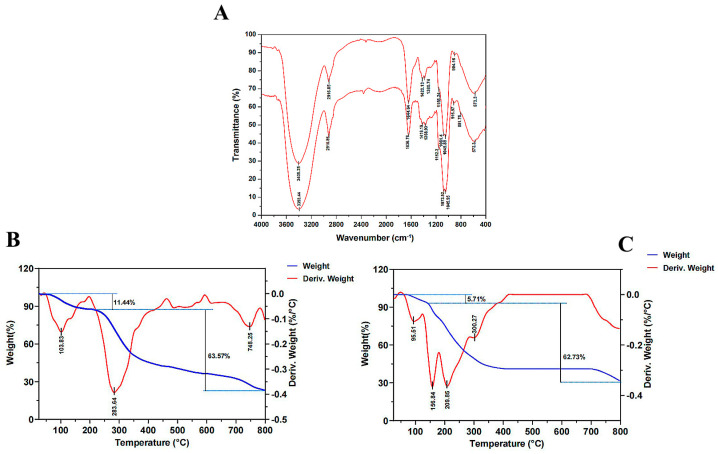
Structure analysis of SDF and SE-SDF from ginger peel residue: (**A**) FT-IR image; (**B**) TGA image of SDF; (**C**) TGA image of SE-SDF.

The FT-IR of SDF and SE-SDF from ginger peel residue is shown in Figure 2A. A broad intense peak in the range of 3393–3405 cm^−1^ represented the stretching vibration of O–H linkage, and the SE-SDF peak position underwent a red shift (3393.44 → 3405.25 cm^−1^), indicating a change in hydrogen bonding and a tendency toward a loose structure [19]. The absorption peak at 2916.85 cm^−1^, 1416–1420 cm^−1^ and 1368–1380 cm^−1^ were attributed to the shear vibration of –CH2– and deformation vibration of C-H with slight peak displacement, indicating a certain change in the side chain structure [41,42]. The peak around 1636–1645 cm^−1^ resulted from bound water. The characteristic peaks of C-O and C-OH vibrations were located at 1152–1156 cm^−1^, 1073–1081 cm^−1^, and 1045–1049 cm^−1^, which indicated that the sample had a typical pyranose structure [43,44]. The absorption peak in the 900–920 cm^−1^ region indicated the presence of β-glycosidic bonds, and SE treatment did not disrupt its configuration [45]. The results showed that SDF and SE-SDF had similar infrared spectra with no new peaks in SE-SDF, while shifts in peak intensity and position indicated that SE treatment altered the structure of SDF from ginger peel residue.

### 3.6. Thermal Stability Analysis

As shown in Figure 2B,C, the TG and DTG curves of SDF and SE-SDF exhibited typical polysaccharide pyrolysis characteristics, mainly divided into three stages: dehydration, pyrolysis, and carbonization. At 25 °C–150 °C, SDF and SE-SDF showed significant weight loss, mainly attributed to the evaporation of water. Among them, the weight loss rate of SE-SDF (5.71%) was significantly lower than that of SDF (11.44%). Within the temperature range of 150 °C–400 °C, polysaccharides underwent the primary pyrolysis stage, accompanied by the glycosidic bond cleavage, dehydration reaction, and molecular chain cleavage, which resulted in pronounced weight loss [46]. Among them, SDF showed the maximum weight loss peak at 283.64 °C, while SE-SDF exhibited multiple weight loss peaks in the range of 156.84 °C–300.27 °C. This phenomenon indicated that SE pretreatment induced the structural reconstruction and compositional changes in polysaccharides. When the temperature exceeded 400 °C, SDF and SE-SDF entered a slow carbonization stage, the final residual rate of SE-SDF (31.50%) was higher than that of SDF (23.08%), indicating that SE-SDF had better thermal stability.

### 3.7. SEM Analysis

**Figure 3:**
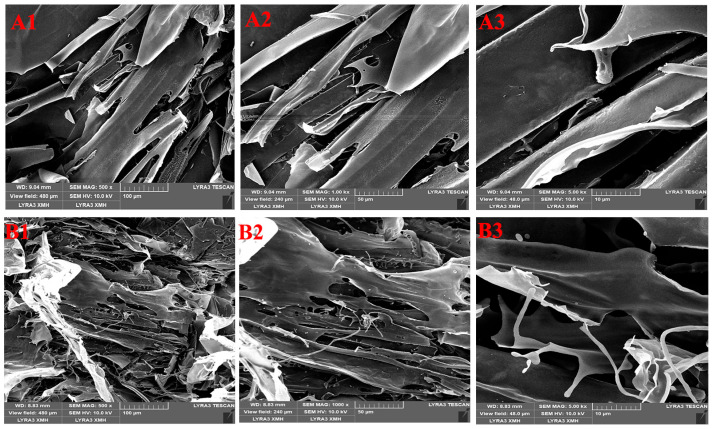
SEM images of SDF and SE-SDF from ginger peel residue: (**A1**–**A3**) SDF; (**B1**–**B3**) SE-SDF; (1) 500×; (2) 1000×; (3) 5000×.

The SEM results of SDF from ginger peel residue before and after SE pretreatment were shown in Figure 3. As shown in Figure 3(A1–A3), the surface of SDF was dense and intact, and had a relatively regular smooth and scaly structure. After SE treatment, the surface of SE-SDF was rough, fluffy, and presented a typical honeycomb-like porous structure (Figure 3(B1–B3)). It may be due to the steam penetrating into the cells and tissues of ginger peel residue by the SE treatment. Upon rapid pressure release, the steam expanded and escaped sharply to generate intense shear forces that disrupted the fibrous structure, resulting in a loosened matrix with increased porosity [47].

### 3.8. Physicochemical Analysis

As shown in Table 5, SE treatment had a significant impact on the physicochemical properties of SDF from ginger peel residue. After SE treatment, the WHC and SC of SE-SDF were significantly increased by 38.89% and 30.77%, respectively. These improvements may be attributed to disruption of the original dense structure by SE treatment, which promoted the dissociation of the fiber components and exposed more hydrophilic groups. Meanwhile, the loose and porous structure formed after SE treatment may provide more water-binding sites and internal spaces, thereby enhancing WHC and SC. Similar structural changes have been reported in wheat bran, SE treatment significantly enhanced the water holding capacity and swelling capacity of SDF [48]. In contrast, the OHC decreased from 14.98 g/g to 13.74 g/g, which was different from the previous studies [49,50]. This reduction may be related to the degradation or rearrangement of hydrophobic structural components, resulting in weakened hydrophobic interactions and decreased oil retention ability [51].

### 3.9. Effects of SDF and SE-SDF on HepG2 Cell Viability, TG and TC Content

**Figure 4:**
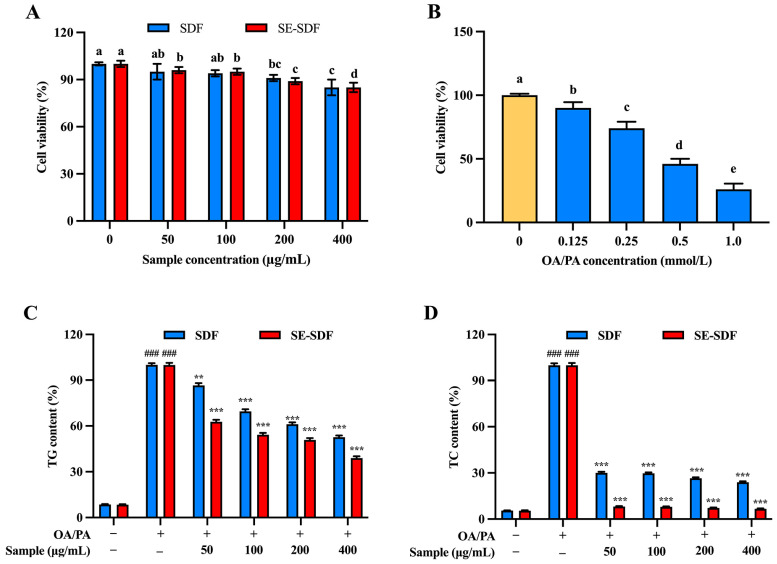
Effect of SDF and SE-SDF from ginger peel residue on hypolipidemic effect of HepG2 cells: (**A**) cell viability of SDF and SE-SDF; (**B**) cell viability of OA/PA; (**C**) TG content; (**D**) TC content. **Note:** Different letters indicate significant differences among groups (*p* < 0.05). # *p* < 0.05, ## *p* < 0.01, ### *p* < 0.001 compared with the control group, * *p* < 0.05, ** *p* < 0.01, *** *p* < 0.001 compared with the OA/PA group.

As shown in Figure 4A, no significant cytotoxicity was observed after treatment with SDF or SE-SDF at concentrations between 50 and 400 µg/mL, with cell viability remaining above 80%. These concentrations were therefore selected for subsequent experiments. In contrast, OA/PA exposure resulted in a gradual decline in HepG2 cell viability (Figure 4B). Compared with the blank control group, the OA/PA treatment decreased the cell viability in a dose-dependent manner, indicating that OA/PA had a toxic effect on cells but could still be used. When the concentration was higher than 0.125 mmol/L, the cell viability significantly decreased to 89% (*p* < 0.05). Therefore, an OA/PA concentration of 0.125 mmol/L was used to establish a hyperlipidemia damage model.

The effects of SDF and SE-SDF on HepG2 cells are shown in Figure 4C,D. Compared with the control, the treatment with OA/PA significantly (*p* < 0.05) increased the TG and TC content, confirming the successful establishment of the OA/PA-induced HepG2 cells model. Compared with the model group, both SDF and SE-SDF of ginger peel residue significantly suppressed the increase in TG content in a dose-dependent manner, with the strongest effects observed at 400 µg/mL (suppressed by 47.46% and 61.02%, respectively). Similarly, the SDF and SE-SDF at 50, 100, 200, or 400 µg/mL significantly suppressed the increase in TC level. Although there was no significant difference between different doses, the inhibitory effect increased with increasing concentration, and the highest inhibition rate was observed at a dose of 400 μg/mL (76.0% and 93.3%, respectively). A previous study on tea residue also reported strong hypolipidemic effects of SDF and SE-SDF [52], consistent with the results of this study.

### 3.10. Effects of SDF and SE-SDF on Zebrafish Model

**Figure 5:**
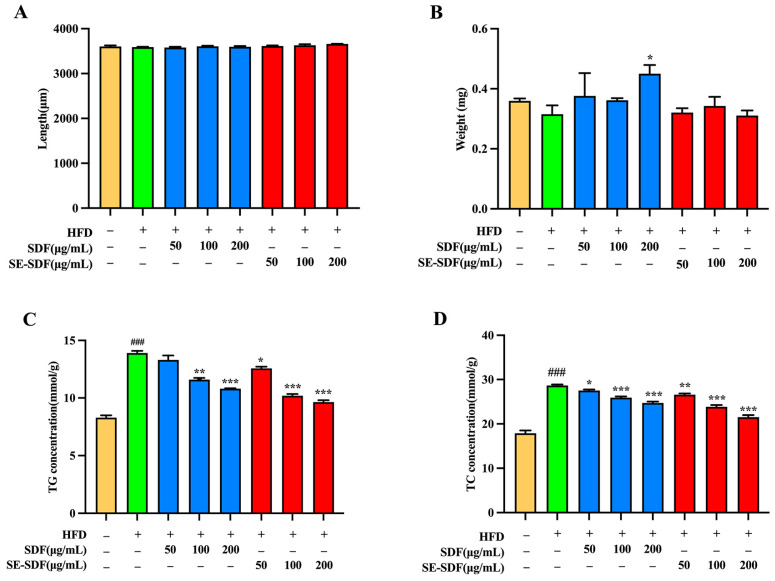
Effect of SDF and SE-SDF from ginger peel residue on hypolipidemic effect in zebrafish: (**A**) body length; (**B**) weight; (**C**) TG concentration; (**D**) TC concentration. Note: # *p* < 0.05, ## *p* < 0.01, ### *p* < 0.001 compared with the control group, * *p* < 0.05, ** *p* < 0.01, *** *p* < 0.001 compared with the OA/PA group. The different colors represent different treatment groups.

TG and TC are key indicators of lipid metabolism status. Under normal physiological conditions, blood lipids are maintained at a relatively stable range, whereas lipid metabolic disorders are commonly accompanied by abnormal changes in TG and TC levels [53]. The effects of SDF and SE-SDF from ginger peel residue on body length, weight, TG and TC content are shown in Figure 5. As shown in Figure 5A, there was no significant difference in zebrafish body length between different treatment groups (*p* > 0.05). Compared with the model group, zebrafish treated with 200 µg/mL SDF exhibited a significantly higher body weight (*p* < 0.05), whereas no significant differences were detected in the remaining treatment groups (Figure 5B).

Figure 5C,D showed that HFD group increased the TG and TC contents compared with the control group (*p* < 0.05), indicating that the zebrafish hyperlipidemia model was successfully established. SDF and SE-SDF at 50, 100, and 200 µg/mL effectively suppressed the HFD-induced elevations in TG and TC levels. Among the tested doses of the SDF and SE-SDF from ginger peel residue, 200 µg/mL showed the greatest hypolipidemic effect. At this concentration, SDF and SE-SDF reduced TG levels by 22.30% and 30.58%, respectively, and reduced TC levels by 13.72% and 24.89%, respectively. These results indicated that the SE-SDF exhibited stronger hypolipidemic effect than SDF. Similar hypolipidemic activity of SDF were reported previously. For example, the SDF and SE-SDF from *Flammulina velutipes* can significantly reduce the TG and TC levels in zebrafish, with SE-SDF showing superior efficacy [19]. The above results suggested that SE treatment enhances the hypolipidemic activity of SDF from ginger peel residue.

### 3.11. Effects of SDF and SE-SDF on the Lipid Accumulation in the Caudal Vasculature and Liver Regions of Zebrafish

**Figure 6:**
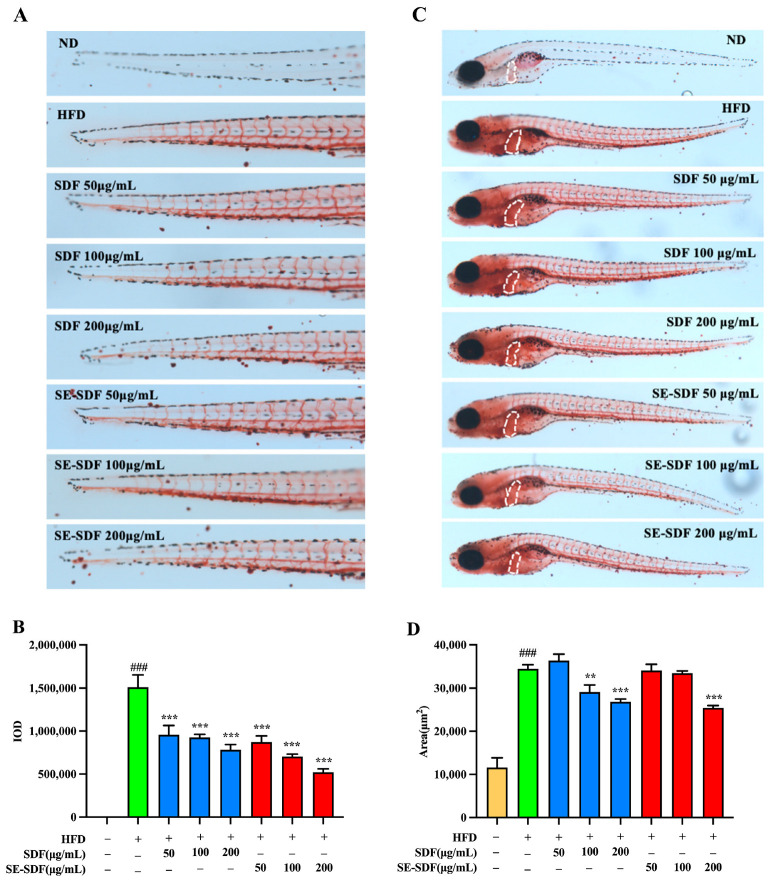
Effect of SDF and SE-SDF from ginger peel residue on hypolipidemic effect in zebrafish: (**A**) staining of caudal vasculature; (**B**) IOD of caudal vasculature; (**C**) staining of liver (white dashed lines); (**D**) staining area of liver. **Note:** # *p* < 0.05, ## *p* < 0.01, ### *p* < 0.001 compared with the control group, * *p* < 0.05, ** *p* < 0.01, *** *p* < 0.001 compared with the HFD group. The different colors represent different treatment groups.

Lipid metabolism disorders are typically characterized by abnormal lipid accumulation in tissues and vessels. To evaluate the effects of different treatments on lipid accumulation in zebrafish, Oil red O staining was used to observe and analyze the lipid deposition in caudal vasculature. As shown in Figure 6A,B, no significant lipid deposition was observed in the caudal vessels of zebrafish in the ND group, whereas a large amount of orange-red lipid accumulation was detected in the HFD group, indicating that the high-fat treatment markedly promoted vascular lipid deposition. Notably, the treatment with SDF and SE-SDF significantly suppressed the lipid deposition of caudal vasculature in a dose-dependent manner (*p* < 0.05). The most effective dose of the SDF and SE-SDF for such suppressions was 200 μg/mL, with SDF and SE-SDF reducing vascular lipid deposition by 48.30% and 65.59%, respectively. Lipid deposition in liver regions is further analyzed in Figure 6C,D; HFD treatment increased hepatic lipid deposition 2.62-fold. Among the tested concentrations, 200 µg/mL SDF and SE-SDF showed the greatest hypolipidemic effect, reducing hepatic lipid deposition by 32.12% and 33.86%, respectively. This result was consistent with the results of lipid deposition in caudal vasculature, further verifying that SE modification enhanced the hypolipidemic activity of SDF from ginger peel residue. Taken together, SDF and SE-SDF alleviated HFD-induced lipid metabolic disorder in zebrafish by reducing TG and TC levels and suppressing lipid deposition in both the caudal vasculature and liver, with SE-SDF showing superior protective effects. However, the underlying molecular mechanisms remain unclear and require further investigation using proteomics and metabolomics approaches.

## 4. Conclusions

In this study, a hypolipidemic SDF extract was obtained from ginger peel residue using SE-assisted extraction. The optimal SE extraction conditions were established through single-factor and orthogonal experiments as follows: material-to-cavity ratio of 2:5; SE pressure, 1.5 MPa; and SE time, 300 s. Under these conditions, the extraction rate of SDF reached 8.20%, representing a 210.60% increase compared with the untreated group. Monosaccharide composition and Mw results showed that the content of galactose, xylose, and arabinose in SE-SDF increased by 12.22%, 164.22%, and 59.79%, respectively. And the Mw of SE-SDF was markedly lower than that of SDF, indicating improved solubility and reduced viscosity. In addition, SE-SDF exhibited superior thermal stability and hydration properties. In OA/PA-stimulated HepG2 cells, both SDF and SE-SDF at 50–400 µg/mL could significantly reduce the levels of TG and TC (*p* < 0.05), with SE-SDF showing stronger inhibitory activity than SDF. In HFD-induced zebrafish model, 200 µg/mL SE-SDF significantly reduced TG and TC levels and decreased the lipid deposition in caudal vasculature and liver. However, the molecular mechanism underlying its hypolipidemic activity remains to be further clarified. Future research will investigate the mechanism of SE-SDF by integrating transcriptomic and metabolomic analyses and further explore its application in functional food products and dietary supplements.

## Figures and Tables

**Table 1:** Orthogonal experimental factors and levels.

Level	Factor		
	A	B	C
1	1:5	1.2	180
2	2:5	1.5	240
3	3:5	1.8	300

**Table 2:** Orthogonal experimental results and analysis.

Run	Factor			Extraction Rate (%)
	A	B	C	
1	1:5	1.2	180	7.76
2	1:5	1.5	240	7.98
3	1:5	1.8	300	7.74
4	2:5	1.2	240	7.91
5	2:5	1.5	300	8.12
6	2:5	1.8	180	7.61
7	3:5	1.2	300	7.66
8	3:5	1.5	280	7.71
9	3:5	1.8	240	7.62
k_1_	7.827	7.777	7.693	
k_2_	7.880	7.937	7.837	
k_3_	7.663	7.657	7.840	
R	0.217	0.280	0.147	

**Table 3:** The chemical components of SDF and SE-SDF from ginger peel residue.

Sample	Total Polysaccharide (%)	Neutral Polysaccharide (%)	Acidic Polysaccharide (%)	Protein (%)
SDF	93.27 ± 1.17 ^a^	85.36 ± 0.98 ^b^	7.91 ± 0.43 ^a^	0.38 ± 0.02 ^b^
SE-SDF	94.69 ± 0.48 ^a^	89.29 ± 1.46 ^a^	5.40 ± 0.36 ^b^	1.49 ± 0.08 ^a^

**Note:** Different letters indicate significant differences among groups (*p* < 0.05).

**Table 4:** The monosaccharide composition and Mw of SDF and SE-SDF from ginger peel residue.

Sample	Glucose (%)	Galactose (%)	Xylose (%)	Arabinose (%)	GalUA (%)	Mw (kDa)
SDF	90.36	3.11	1.09	2.86	0.03	330.89
SE-SDF	89.05	3.49	2.88	4.57	-	122.99

**Note:** Mw represents weight average molecular weight.

**Table 5:** The physicochemical properties of SDF and SE-SDF from ginger peel residue.

Sample	WHC (g/g)	OHC (g/g)	SC (mL/g)
SDF	0.36 ± 0.13 ^b^	14.98 ± 0.11 ^a^	0.13 ± 0.04 ^b^
SE-SDF	0.50 ± 0.11 ^a^	13.74 ± 0.13 ^b^	0.17 ± 0.04 ^a^

**Note:** Different letters indicate significant differences among groups (*p* < 0.05).

## Data Availability

The original contributions presented in the study are included in the article, further inquiries can be directed to the corresponding authors.

## Abbreviations

ABTS, 2,2′-Azino-bis(3-ethylbenzothiazoline-6-sulfonic acid; ANOVA, analysis of variance; CCK-8, cell counting Kit-8; DF, dietary fiber; DPPH, 2,2-diphenyl-1-picrylhydrazyl; FT-IR, Fourier infrared spectroscopy; HFD, high-fat diet; HPLC, high-performance liquid chromatography; HPGPC, high-performance gel permeation chromatography; IDF, insoluble dietary fiber; Mw, molecular weight; OA/PA, oleic acid/palmitic acid; OHC, oil holding capacity; SC, swelling capacity; SE, steam explosion; SEM, scanning electron microscopy; SDF, soluble dietary fiber; TC, total cholesterol; TG, triglycerides; TGA thermogravimetric analysis; 15 dpf, 15 days post fertilization.
